# Population Structure of a Hybrid Clonal Group of Methicillin-Resistant *Staphylococcus aureus*, ST239-MRSA-III

**DOI:** 10.1371/journal.pone.0008582

**Published:** 2010-01-05

**Authors:** Davida S. Smyth, Linda K. McDougal, Frode W. Gran, Anand Manoharan, Mark C. Enright, Jae-Hoon Song, Herminia de Lencastre, D. Ashley Robinson

**Affiliations:** 1 Department of Microbiology and Immunology, New York Medical College, Valhalla, New York, United States of America; 2 Office of Antimicrobial Resistance, Centers for Disease Control and Prevention, Atlanta, Georgia, United States of America; 3 Department of Medical Microbiology, University Hospital Trondheim, Trondheim, Norway; 4 Department of Medicine Unit I and Infectious Diseases, Christian Medical College, Vellore, India; 5 Department of Infectious Disease Epidemiology, Imperial College London, London, United Kingdom; 6 Division of Infectious Diseases, Samsung Medical Center, Sungkyunkwan University School of Medicine, Seoul, Korea; 7 Laboratory of Molecular Genetics, Instituto de Tecnologia Química e Biológica, Universidade Nova de Lisboa, Oeiras, Portugal; 8 Laboratory of Microbiology, The Rockefeller University, New York, New York, United States of America; National Institute of Allergy and Infectious Diseases, National Institutes of Health, United States of America

## Abstract

The methicillin-resistant *Staphylococcus aureus* (MRSA) clonal group known as ST239-MRSA-III is notable for its hybrid origin and for causing sustained hospital epidemics worldwide since the late 1970s. We studied the population structure of this MRSA clonal group using a sample of 111 isolates that were collected over 34 years from 29 countries. Genetic variation was assessed using typing methods and novel ascertainment methods, resulting in approximately 15 kb of sequence from 32 loci for all isolates. A single most parsimonious tree, free of homoplasy, partitioned 28 haplotypes into geographically-associated clades, including prominent European, Asian, and South American clades. The rate of evolution was estimated to be approximately 100× faster than standard estimates for bacteria, and dated the most recent common ancestor of these isolates to the mid-20th century. Associations were discovered between the ST239 phylogeny and the *ccrB* and *dru* loci of the methicillin resistance genetic element, SCC*mec* type III, but not with the accessory components of the element that are targeted by multiplex PCR subtyping tools. In summary, the evolutionary history of ST239 can be characterized by rapid clonal diversification that has left strong evidence of geographic and temporal population structure. SCC*mec* type III has remained linked to the ST239 chromosome during clonal diversification, but it has undergone homoplasious losses of accessory components. These results provide a population genetics framework for the precise identification of emerging ST239 variants, and invite a re-evaluation of the markers used for subtyping SCC*mec*.

## Introduction

As one of the most common antibiotic-resistant bacterial pathogens, methicillin-resistant *Staphylococcus aureus* (MRSA) pose immense challenges to healthcare systems throughout the world [1]. During the past decade, studies have provided a coarse outline of the population structure of this pathogen [2]–[4]. Five clonal groups have spawned the majority of nosocomial MRSA that are isolated worldwide, and they can be readily identified by their founding multilocus sequence types (ST): ST5, ST8, ST22, ST30, and ST45 [2]. It was deduced that the staphylococcal chromosomal cassette *mec* (SCC*mec*) genetic element, which confers the methicillin resistance phenotype [5], has been horizontally transferred into these five clonal groups on many occasions, giving rise to numerous individual MRSA clones [6], [7]. Recent work provided a fine-scale outline of the population structure of one clonal group, ST5, and extrapolated that most of the individual clones that arise within MRSA clonal groups have a relatively limited ability to spread geographically [6]; that is, SCC*mec* acquisitions in local populations are more frequent events than global migrations of MRSA clones. However, decades of molecular epidemiological studies suggest that the hybrid MRSA clonal group, ST239, has spawned multiple globally disseminated variants that occur with a single SCC*mec* type. Do different MRSA clonal groups have different population structures?

ST239 was the first bacterial hybrid to be identified in nature [8]. Its chromosome has estimated parental contributions of approximately 20% and 80% from distantly related ST30- and ST8-like parents, respectively. While horizontal transfer and recombination of genetic material occurs widely in bacteria [9], the salient observation for ST239 was that the recombined genetic material occurred as large chromosomal replacements rather than many localized recombinations [8]. Bacterial hybrids of this sort have been identified recently among *Streptococcus agalactiae* [10]. In addition, hybrids identified from multilocus sequence typing data have been reported among *Campylobacter jejuni*, *Escherichia coli*, and *Vibrio vulnificus* [11]–[13]. An emerging theme from these studies is that bacterial hybrids can represent clinically significant pathogens.

Some of the earliest published reports of MRSA infections that are known to be caused by ST239 are of hospital epidemics of phage group III, gentamicin-resistant, MRSA from Australia, the UK, and the USA, that began during the late 1970s and early 1980s [14]–[16]. Hospital epidemics caused by ST239 are documented throughout Europe and South America during the 1980s and 1990s [17]–[25], and throughout Asia and the Middle East during the 1990s and 2000s [26]–[37]. Currently, ST239 is a major cause of MRSA infections in Asian hospitals; one study suggested that ST239 accounts for 90% of the nosocomial MRSA infections in a geographic region that holds 60% of the world's human population [38]. ST239 has been reported recently in the UK as an outbreak cause of bacteremia from vascular access devices [39].

Pulsed-field gel electrophoresis (PFGE) has shown that extensive variation can be generated in the ST239 chromosome over years of isolation from a local population, and that a single PFGE type usually predominates in any given local population [19], [21], [25], [27], [31], [40]. As ST239 has diversified into a clonal group and spread geographically, it has been given more than a dozen names including: the Brazilian [24], British Epidemic (EMRSA-1, -4, -7, -9, -11) [41], Canadian Epidemic (CMRSA-3)/Punjab [42], Czech [21], Eastern Australian (AUS-2, -3) [43], Georgian [29], Hungarian [20], Lublin [23], Nanjing/Taipei [26], Portugese [22], and Vienna [44] clones. In contrast to the high levels of PFGE variation displayed by ST239, nearly all of its isolates carry the SCC*mec* type III genetic element, which is a composite element of approximately 67 kb that confers resistance to methicillin and other antimicrobials. Reports of methicillin-susceptible isolates of ST239 and resistant isolates with non-III SCC*mec* types are rare. Prevalent variations in SCC*mec* type III are attributed to the Brazilian and Portugese variants, types IIIA and IIIB [45], respectively. However, essentially nothing is known about the evolutionary relationships between ST239 variants or the details of their relationships with SCC*mec* type III.

The goal of this study was to obtain a fine-scale outline of the population structure of ST239. We sought to reconstruct ST239's phylogeny in order to address several basic questions: (i) have large chromosomal replacements continued to occur within this clonal group, (ii) does this clonal group display spatiotemporal population structure, (iii) has SCC*mec* type III been acquired by this clonal group on multiple occasions?

## Materials and Methods

### Bacterial isolates

The study sample consisted of 111 isolates of ST239 and closely related variants, selected from multiple isolate collections to maximize genetic, geographic, and temporal diversity. Most isolates were recognized as members of the ST239 clonal group by previous characterization with PFGE, multilocus sequence typing (MLST), or *spa* typing. The isolates were from 29 countries and spanned 34 years of isolation (1971–2005). Isolate characteristics are listed in Table S1. For this study, long-term storage of isolates was at −80°C and routine growth was done overnight on tryptone soya agar plates at 37°C.

### Identification of genetic variation

Genetic variation was identified for phylogenetic reconstruction using two typing methods and two ascertainment methods. The two typing methods included MLST [46] and *S. aureus* surface protein-encoding gene typing (SAS) [7]. MLST defines the ST239 clonal group based on sequences at seven housekeeping loci, whereas SAS uses sequences at seven loci that encode putative or proven surface proteins. Typing data from these 14 loci were completed for all 111 isolates.

The two ascertainment methods both involved an initial screen for genetic variation at many loci for a subset of 10 diverse isolates, called the discovery panel of isolates (Figure S1), followed by sequencing of the variable loci for all 111 isolates. PCR primers for the variable loci are listed in Table S2. The first ascertainment method was direct sequencing of PCR amplicons from 28 loci previously used to pinpoint the junctions of the large chromosomal replacements of ST239 (PCR screen) [8]. This PCR screen resulted in 13 variable loci for which sequences were obtained for all 111 isolates. The second ascertainment method was reduced representation shotgun sequencing (RRS) [47]. With RRS, the genetic variation among >77 loci was found in libraries that were made from pooled DNA of the discovery panel of isolates. To our knowledge, this study represents the first application of RRS to a prokaryote. Details of the RRS procedure are described in Text S1.

### Characterization of SCC*mec* type III

The presence of SCC*mec* type III was confirmed in all isolates using PCR with primers that detect *ccrAB3* and class A *mec* [5], [7]. Since SCC*mec* type III is a composite element that consists of SCC*mec* and an independent element called SCC*mercury* [48], we separately screened for SCC*mercury* with primers that detect *ccrC* [49]. SCC*mec* type III variants were characterized using a multiplex PCR method that has been validated for detecting structural variations of type IIIA, which has lost the pT181 integrated plasmid, and type IIIB, which has lost pT181 and SCC*mercury* [45]. A fourth, unnamed variant from Ireland has been characterized with the multiplex PCR method and with further work [50]. Although other SCC*mec* type III variants are known [23], [51], they have not been fully characterized with the multiplex PCR method.

As an alternative subtyping method, we sequenced two regions of SCC*mec* type III. A portion of the *ccrB* gene was sequenced using the primers of Lina et al. [52], and alleles were trimmed to 410 bp. The direct repeat units (*dru* repeats) located in the hypervariable region of SCC*mec* were sequenced using the reverse primer of Tohda et al. [53] and a forward primer of 5′-ACTATTCCCTCAGGCGTCA. *dru* repeat profiles and types were assigned according to the recently proposed scheme of Goering et al. [54], using DruID software available at the *dru* typing server (http://www.dru-typing.org).

### Data analyses

Haplotype and SCC*mec* diversities were measured with a bias-corrected estimator, *k_e3_*, of the effective number of types as described by Nielsen et al. [55]. *k_e3_* estimates the number of equally frequent types that will produce the observed diversity. The variance of *k_e3_*, Var(*k_e3_*), was calculated according to Nielsen et al. [55] and was used to construct 95% confidence intervals with the formula: *k_e3_*+/−2 √Var(*k_e3_*).

Due to the close genetic relationships among the isolates studied here, phylogenetic analyses were done within an unweighted maximum parsimony (MP) framework [56]. Two insertion deletion polymorphisms (indels) were processed as follows: they were aligned based on translated amino acids, collapsed to single variable sites, and treated as fifth character states in subsequent analyses. All variable sites from the sequence alignment were used in MP analysis. PAUP* v4.0b10 software [57] was used to find the most parsimonious tree heuristically, with 100 random taxon addition replicates and tree-bissection-reconnection branch-swapping. Tree length skewness, as measured by the *g1* statistic for 100,000 randomly generated trees, was used to assess the phylogenetic signal in the data [58]. In addition, non-parametric bootstrapping was done using 1000 bootstrap replicates.

Population structure was studied using parsimony and subpopulation differentiation approaches. The parsimony approach was based on the Slatkin-Maddison (SM) test for gene flow [59] and was performed with MacClade v4.08 software [60]. The subpopulation differentiation (SD) approach was based on Hudson et al.'s [61] statistic, *K_ST_*, and was performed with DnaSP v5.00 software [62]. Further study of temporal population structure made use of regression analyses between tree root-to-haplotype distances and haplotype dates of isolation [63], as implemented by Path-O-Gen v1.1 software (by A. Rambaut; http://tree.bio.ed.ac.uk/software/pathogen). Details of these analyses are described in Text S1.

### Nucleotide sequences

Allele sequences have been deposited in GenBank with accession numbers GU084342–GU084380. Unique MLST and *dru* alleles have been deposited in publicly available databases at saureus.mlst.net and www.dru-typing.org.

## Results

### Genetic variation and haplotype phylogeny

In total, 14,996 bp of sequence from 32 loci was obtained for all 111 isolates (Table 1). The combined dataset yielded 43 variable nucleotide sites including 11 synonymous changes and 26 non-synonymous changes in coding regions, 4 changes in intergenic regions, and 2 indels of 9 bp and 90 bp in length. Thirteen of the variable sites were parsimony-informative. The ascertained loci revealed significantly more variable sites (described in Text S1) and more haplotype diversity (*k_e3_*, *P*<0.05) than the typing loci (Table 1). The combined dataset defined 28 unique haplotypes.

**Table 1:** Summary of genetic variation detected by different datasets.

Dataset category	Dataset subcategory	No. loci screened	No. loci selected	Total sequence (bp)	No. variable sites	No. informative sites	No. haplotypes	*k_e3_* (95% C.I.)
Typing	MLST	7	7	3198	7	1	7	1.3 (0.9, 1.8)
	SAS	7	7	3101	4	1	5	1.5 (1.3, 1.8)
Ascertainment	PCR screen	28	13	7308	26	10	18	4.3 (2.4, 6.2)
	RRS	77+	5	1389	6	1	6	2.3 (1.9, 2.6)
Combined		119+	32	14996	43	13	28	7.9 (4.2, 11.6)

Acronyms: multilocus sequence typing (MLST), *Staphylococcus aureus* surface protein-encoding gene typing (SAS), previously studied loci (PCR screen), reduced representation shotgun sequencing (RRS).

77 multiseq contigs were counted as screened.

*k_e3_* is a biased-corrected estimator of the effective number of haplotypes.

**Figure 1:**
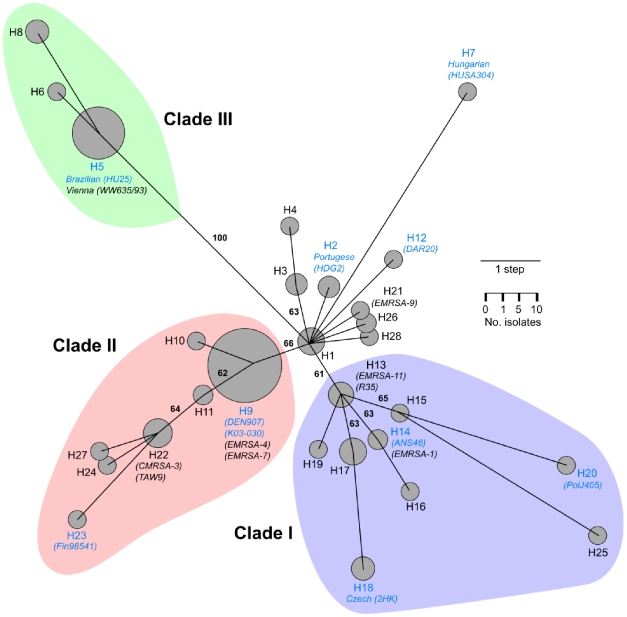
Evolutionary relationships within the ST239 clonal group. Tree is the most parsimonious haplotype tree found by heuristic search. Haplotypes are numbered H1–H28. Circles represent the number of isolates of each haplotype. Names of important strains are indicated in parentheses below select haplotypes. Blue labels indicate the discovery panel of isolates. Numbers along branches are bootstrap proportions. Three prominent clades are highlighted.

A single most parsimonious haplotype tree was found (Figure 1), which had a length of 44 steps and a consistency index of 1.0. Since no homoplasy was observed at the 32 loci, the data indicated that ST239 has diversified clonally via point mutations. No branch collapse artifact due to ascertainment was evident on the tree; the tree pathways between the nine haplotypes of the discovery panel of isolates all contained secondary branches (Figure 1, blue haplotype labels). Significant non-random phylogenetic signal was present in the data under the parsimony criterion, as the shortest random tree out of 100,000 examined random trees was 17 steps longer than the observed tree (*g1* statistic, *P*<0.05). Moreover, all internal nodes on the tree received >60% bootstrap support (Figure 1).

Three prominent clades emanated from the central H1 haplotype (Figure 1, labeled I–III). In addition, a minor clade (H3, H4) and six other haplotypes (H2, H7, H12, H21, H26, H28) connected to H1. Interestingly, the oldest ST239 isolates that we have located thus far were isolated in 1971 from Norway and have the H1 haplotype. These isolates are a decade older than one of the oldest published isolates of ST239, strain ANS46 isolated in 1982 from Australia [15], which has the H14 haplotype. Rooting of the tree based on both outgroup and molecular clock criteria indicated that the ST8-like portion of the chromosome was rooted at H1, whereas the ST30-like portion of the chromosome was rooted at the adjacent H13 haplotype. These results indicated that the root of the tree should be placed at the central H1 haplotype or along the branch connecting H1 and H13.

### Statistical phylogeographic associations

**Figure 2:**
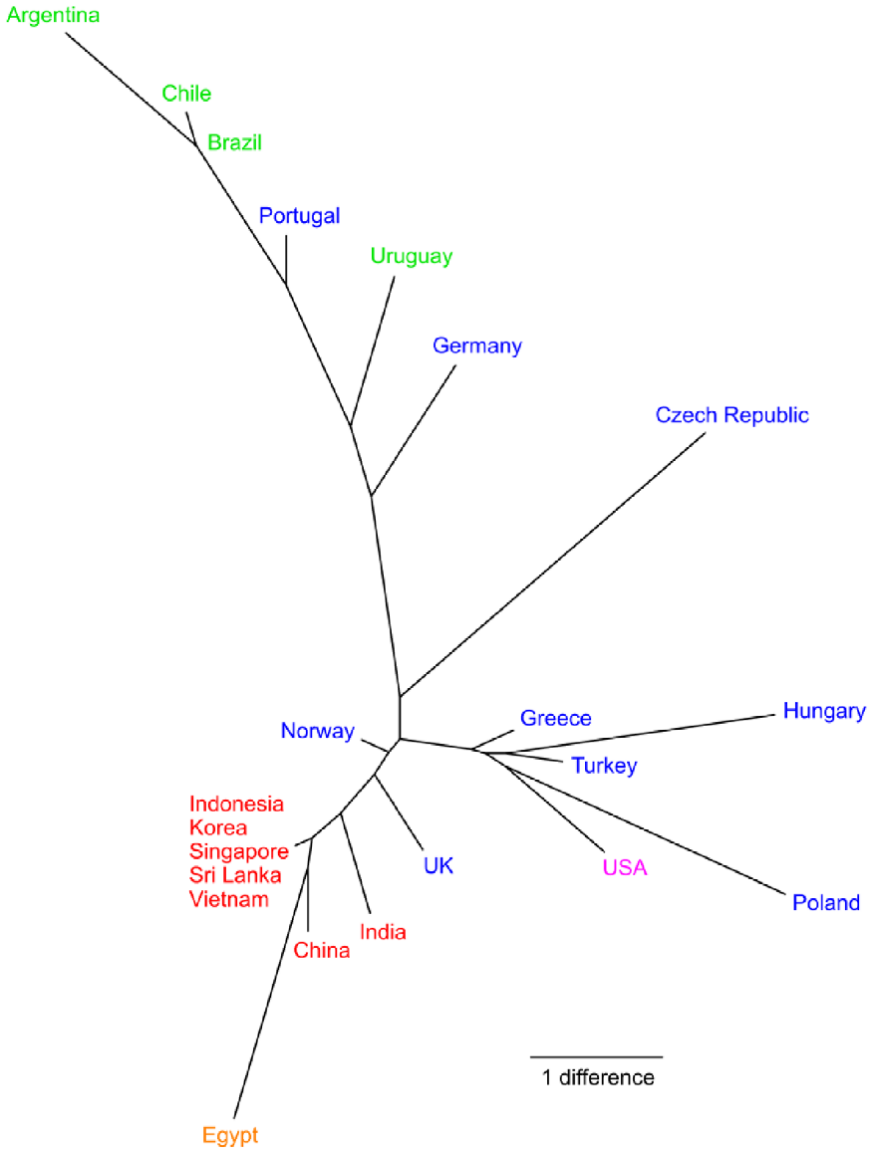
Geographic structure within the ST239 clonal group. Tree is a neighbor-joining population tree based on the average number of pairwise nucleotide differences between countries. Labels are colored according to continents.

Analyses based on the SM and SD tests provided strong statistical support for geographic population structure (Table 2). Structuring by continents tended towards statistical significance (0.05<*P*<1.0) based on the SM test, and it achieved high significance based on the SD test. Furthermore, structuring by countries was highly significant with both tests (Table 2). A population tree constructed from the average number of pairwise nucleotide differences between countries clearly depicted a geographic structure of countries clustering by continents (Figure 2). These results provided evidence that ST239 variants tend to spread locally rather than globally, similar to the findings of the ST5 clonal group [6]. However, we note that five haplotypes (H5, H9, H13, H14, H22) were isolated from multiple continents and may therefore represent highly transmissible variants.

**Table 2:** Analyses of population structure using clone-corrected datasets.

		Slatkin-Maddison (SM) tests					Subpopulation differentiation (SD) tests			
Character or population category	Character or population subcategory	Character type	No. character states	No. taxa	*S_RR_*	*P*	No. sub-populations	No. taxa	*K_ST_*	*P*
Geography	Continents	Unordered	5	36	13.7	0.098	5	33	0.152	<0.001
	Countries	Unordered	5	57	16.4	<0.001	16	42	0.244	0.002
Time	Date of isolation	Ordered	21	53	351.1	0.001	-	-	-	-
SCC*mec*	multiplex PCR	Unordered	9	40	21.6	0.430	7	38	0.072	0.065
	pT181	Unordered	2	35	11.2	0.572	2	35	0.017	0.087
	SCC*mercury*	Unordered	2	31	6.4	0.429	2	31	0.004	0.337
	*ccrB* sequences	Unordered	4	32	4.8	0.001	3	31	0.221	<0.001
	*dru* sequences	-	-	-	-	-	9	24	0.274	<0.001
	*dru* lengths	Unordered	13	45	24.4	<0.001	8	40	0.110	0.015

SM tests generally had more taxa per category because all available haplotypes were included, whereas subpopulations that consisted of single haplotypes were not included in SD tests.

For SM tests, the 29 countries were analyzed as 5 continents weighted by country; one of each haplotype from each country was used. The 42 *dru* sequences were too diverse to be used with MacClade.

For SD tests, time's ordered character type was not applicable.

Closer inspection of geographic sources of isolation revealed that clade I was predominantly European isolates (19/22 isolates), clade II was predominantly Asian isolates (34/45 isolates), and clade III was predominantly South American isolates (13/25 isolates); clade III also contained a high proportion of European isolates from Portugal (8/10 isolates) (Table S1). The remaining haplotypes, connected to the central H1 haplotype, were also predominantly European isolates (12/19 isolates). Sampling of exclusively European isolates from the central H1 haplotype suggested a European origin for the ST239 clonal group. Consistent with this interpretation, European isolates presented more haplotype diversity (*k_e3_*, *P*<0.05) than Asian and South American isolates. However, South American isolates were not sampled as well as Asian and European isolates (15, 39, and 46 isolates, respectively), and African, Australian, and North American isolates were poorly sampled (5, 1, and 3 isolates respectively), so additional sampling is necessary to determine the geographic origin of this clonal group.

### Evidence of rapid evolution and a recent origin

The presence of old isolates at internal nodes on the tree suggested that their evolution could possibly be measured through time. Treating time as an ordered, irreversible character, the SM test provided strong statistical support for temporal population structure (Table 2). Furthermore, regression analyses showed that the amount of genetic variation accumulated by haplotypes (i.e. tree root-to-haplotype distances) was significantly correlated with their dates of isolation (Table 3). This proof of temporal signal in the data justifies attempts to estimate the rate of evolution and date of the most recent common ancestor (MRCA) from the data; it suggests that the ST239 clonal group is measurably evolving [64]. Similar rate and date estimates were obtained from the ST8- and ST30-like parental portions of the ST239 chromosome and from jackknife and crude estimates (Table 3). This level of concordance [65] supported the notion that the sampled ST239 isolates shared a single common ancestor.

**Table 3:** Estimates of rate of evolution and date of the most recent common ancestor (MRCA).

		Correlation		Rate of evolution		Date of MRCA (year)	
Chromosome region	Ascertainment-adjusted sequence length (bp)	*r*	*P*	Crude estimate	Jackknife estimate (95% C.I.)	Crude estimate	Jackknife estimate (95% C.I.)
ST8-like	12,419	0.358	0.008	4.8	3.4 (0, 7.0)	1955.5	1945.2 (1919.1, 1971.2)
ST30-like	9,500	0.355	0.009	4.4	3.1 (0, 7.3)	1963.0	1961.8 (1948.6, 1975.0)

Correlation is from a regression analysis of tree root-to-haplotype distances versus haplotype dates of isolation. Estimates of the rate of evolution and date of the MRCA are made from the slope and x-intercept, respectively, of the regression line.

Rate of evolution is in units of 10^−6^ nucleotide changes per site per year.

Remarkably, the rate of evolution was 3.3 to 4.6×10^−6^ nucleotide changes per site per year for averaged jackknife and crude estimates, respectively (Table 3). This rate was much closer to the 3.2 to 4.6×10^−5^ estimates recently reported from *C. jejuni*, *Helicobacter pylori*, and *Neisseria gonorrhea* [12], [66], [67] than to the standard 3×10^−8^ estimate from *E. coli* [68] that was used to calibrate a molecular clock for the ST5 clonal group [6]. The rate estimated here is best characterized as an absolute mutation rate rather than a substitution rate because most of the changes are non-synonymous and non-informative and have not yet been purged through purifying selection. Following the rate of evolution back in time, we found that the date of the MRCA was 1953.5 to 1959.3 for averaged jackknife and crude estimates, respectively (Table 3). These estimates indicated a mid-20th century origin for the MRCA of the sampled isolates, which falls within an important period of time in the history of *S. aureus* - the clinical introductions of penicillin in 1941 and penicillinase-resistant β-lactams such as methicillin in 1959.

### Diversification of the SCC*mec* type III methicillin resistance element

We used the ST239 phylogeny to investigate the diversification of SCC*mec* type III and to obtain insight into the phylogenetic utility of multiplex PCR [45] and sequence [52], [54] methods for subtyping this element. The multiplex PCR method detected nine variants in our study sample, including the four previously characterized types III, IIIA, and IIIB [45], and the unnamed Irish type [50], as well as five newly identified types. The multiplex PCR types are shown in Figure S2. The *ccrB* sequences revealed only four alleles, but the *dru* sequences revealed 42 alleles. These three different typing tools detected significantly different amounts of SCC*mec* type III diversity, with *ccrB* sequences<multiplex PCR<*dru* sequences (Table 4).

**Table 4:** SCC*mec* type III variation as detected by different typing methods.

Method	No. SCC*mec* variants	*k_e3_* (95% C.I.)
multiplex PCR	9	3.5 (2.8, 4.1)
*ccrB* sequences	4	2.3 (2.2, 2.5)
*dru* sequences	42	15.8 (9.0, 22.6)
*ccrB + dru sequences*	45	23.9 (16.3, 31.4)

*k_e3_* is a biased-corrected estimator of the effective number of SCC*mec* variants.

**Figure 3:**
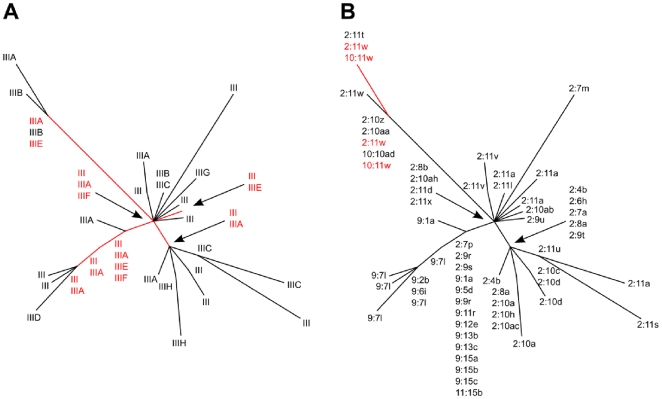
Homoplasy among SCC*mec* type III variants. (A) Mapping of SCC*mec* multiplex PCR types onto the haplotype tree of Figure 1. (B) Mapping of SCC*mec ccrB:dru* sequence types onto the haplotype tree of Figure 1. Red highlights homoplasy where two SCC*mec* subtypes occur together in more than one haplotype.

Population structuring by multiplex PCR types was not statistically significant based on the SM test, but it tended towards significance based on the SD test (Table 2). However, the *K_ST_* value of 0.072 from the SD test provided little evidence of differentiation. The multiplex PCR method targets accessory components of SCC*mec* that are themselves potentially mobile, including pT181 and SCC*mercury* [48]. We recoded the SCC*mec* variants to reflect the presence or absence of pT181 and SCC*mercury* and found no statistical support for population structuring based on the SM or SD tests (Table 2). These results indicated that the multiplex PCR method did not detect variation that generally tracked the ST239 phylogeny. While some individual multiplex PCR types tracked portions of the phylogeny, the most widely distributed types did not (Figure 3A). For example, type IIIH occurred exclusively with two haplotypes (H17, H18) adjacent to each other on the tree, which was consistent with a single origin, but types III and IIIA accounted for 82/111 (74%) of the isolates and each occurred in five haplotypes (H1, H9, H11, H13, H22), which cannot be explained by sampling artifacts or a single origin.

On the other hand, the *ccrB* sequences showed highly significant structuring based on the SM and SD tests, and the *dru* sequences showed highly significant structuring based on the SD test (Table 2). Since the *dru* sequences were too diverse to use with MacClade (i.e. maximum number of character states was 26, number of *dru* alleles was 42), we recoded the sequences to reflect the number of 40 bp *dru* repeats. This recoding resulted in 13 different *dru* alleles (i.e. *dru* lengths) and was amenable to MacClade analysis. Interestingly, statistically significant population structuring by *dru* lengths was noted with the SM and SD tests (Table 2). These results indicated that variation at both the *ccrB* and *dru* loci tracked the ST239 phylogeny, with a single exception (Figure 3B).

Two possible explanations for the homoplasy of multiplex PCR types include multiple horizontal genetic transfers of SCC*mec* (e.g. loss of one element and reacquisition of a different element) or multiple losses of accessory components (e.g. pT181 and SCC*mercury*) from an ancestral type III element. Horizontal genetic transfer should result in a lack of population structuring at all loci on SCC*mec*, whereas loss of accessory components should not affect other loci. Since the *ccrB* and *dru* loci remained consistent with the phylogeny, it is unlikely that the entire element has been lost and reacquired multiple times. These results strongly argue in favor of a clonal diversification process for SCC*mec* type III that is accompanied by repeated loss of accessory components from the element. Such a dynamic is very different from that reported for the ST5 clonal group, where entire SCC*mec* elements are thought to have been imported on many occasions [6].

### Towards a phylogenetic epidemiology of the ST239 clonal group

A subset of the markers examined here may be useful for routine identification of ST239 in locales where this pathogen is emerging. Alleles at the *sasF*, *rrs8*, and *isaA* loci (Table S1) provide diagnostic markers of clades I–III, respectively. Since SCC*mec* multiplex PCR types are essentially randomly distributed across the phylogeny, its markers are not suitable for identifying related variants in situations other than localized outbreaks. For example, SCC*mec* type IIIA is not sufficient information to identify the Brazilian variant because isolates of this type belong to all three clades due to homoplasious losses of pT181. In contrast, the *ccrB* and *dru* loci provide phylogenetically informative markers that can be used to identify related variants. To assess the epidemiological validity of this population genetic framework, we compared our results with published epidemiological data regarding specific migration and diversification events.

- It was concluded previously that the Canadian Epidemic CMRSA-3 variant (haplotype H22) [42] was imported to British Columbia, Canada in 1993 by a patient from Punjab, India [69]. This variant was noted to be highly transmissible [69]. Remarkably, the ancestor (haplotype H11) of the CMRSA-3 variant was represented in our study exclusively by isolates from India. CMRSA-3 and the Indian isolates also shared *ccrB3.9* and *dru* type 7l. Our data lend strong support to the published transmission scenario [69]. Furthermore, our data indicated that this particular variant has migrated to China [26] and Egypt and it has diversified in haplotype and migrated to Europe.
- The Brazilian variant (haplotype H5) [24] was prevalent among multiple hospitals in the Czech Republic from 1996–1997, but it was displaced by a newly identified Czech variant (haplotype H18) by 2000–2002 [21], [70]. Extensive differences (>6 bands) in the PFGE patterns of these two variants indicated that they were not closely related [21] (Figure S1). However, it has been unclear whether the displacement represented a localized diversification of the Brazilian variant or a migration event [21]. Since the Czech and Brazilian variants are widely separated on the tree (Figure 1), the displacement was clearly not a localized diversification of the Brazilian variant. Our data indicated that the displacement was due to rising prevalence of a distinct haplotype whose ancestor (haplotype H17) was already present in Europe in the early 1990s. The Czech variant and ancestral isolates from Germany, Hungary, and Turkey also shared *ccrB3.2* and *dru* type 10a.

## Discussion

Phylogenies of MRSA clonal groups can provide explanatory frameworks for resolving clonal synonymies, distinguishing migration events from diversification events, dissecting the evolution of clinically important traits, and characterizing transmission dynamics. Since MRSA clonal groups are composed of closely related isolates, extraordinary efforts are required to identify enough genetic variation to measure evolution within the groups. In the field of virology, phylogenies for some closely related viral isolates are easier to obtain due to higher mutation rates [64]. However, through use of increasingly greater amounts of sequence data, increasingly finer-scale phylogenies for MRSA clonal groups can be obtained [6], [7]. Our study has provided a proof-of-principle that reduced representation shotgun sequencing (RRS) [47] is a tool that can be used for nucleotide polymorphism discovery with prokaryotes. Although our implementation of RRS was inefficient compared to a simple PCR screen, an economy of scale should be achievable with RRS and all of the data is useable for screening sequenced genomes for polymorphisms.

The large chromosomal replacement described previously for ST239 [8] was reflected in the sequence data obtained here; distinct ST8-like and ST-30 like parent loci constitute the ST239 chromosome. The left and right junctions of the replacement were included among the 13 loci sequenced from the PCR screen dataset, and the recombination breakpoints previously described for strains EMRSA11 and Fin75541 [8] were observed here. No evidence of secondary replacements was found here. The absence of homoplasy from the sequence data provided strong evidence for a clonal diversification process. In turn, this allowed the reconstruction of a single most parsimonious haplotype tree upon which geographic, temporal, and SCC*mec* characters were mapped.

A phylogeographic pattern found previously for the ST5 clonal group [6] was also found here for the ST239 clonal group; haplotypes tend to spread locally rather than globally. However, even with a limited study sample, which was dominated by South American, Asian, and European isolates, 18% (5/28) of the haplotypes had inter-continental distributions. In contrast to the ST5 clonal group, the ST239 clonal group has an intimate association with a single SCC*mec* element that has been maintained and extensively remodeled over the course of its evolutionary history. This association may be due simply to the newness of the relationship, but no evidence of frequent, local acquisitions of SCC*mec* was found. Alternative explanations for this relationship include enhanced barriers to horizontal genetic transfer in ST239, dysfunctional *ccrAB3* alleles that do not permit efficient excision of SCC*mec* type III from ST239, and epistatic interactions that permit only SCC*mec* type III, not other SCC*mec* types, to function in ST239.

The estimated rate of evolution within the ST239 clonal group (∼10^−6^) was slower than recent estimates from *C. jejuni*, *H. pylori*, and *N. gonorrhea* (∼10^−5^) made from more sophisticated Bayesian analyses [12], [66], [67]. Our data provide a rate estimate for a gram-positive bacterial species and contribute to a growing body of evidence that the short-term bacterial mutation rate may be on the order of 100–1000× times faster than the standard *E. coli* rate (∼10^−8^) [68]. The correlation here between tree root-to-haplotype distances and haplotype dates of isolation was statistically significant but not large, which hinted that rate variation might occur within the clonal group. Long branches leading to clade III and to the Hungarian clone (H7) (Figure 1) might point to particularly rapid rates of evolution or long periods of isolation, but these long branches might also be sampling artifacts that could be broken up with additional isolate sampling or polymorphism discovery (i.e. any of the branches connected to H1 could join to these two long branches). We applied a jackknife method to correct for haplotype sampling bias and we artificially lengthened the sequences to adjust for ascertainment bias in attempts to improve the accuracy of the rate and date estimates. Point estimates for the origin of the MRCA of these isolates prior to the isolation of any MRSA is intriguing. Both ST8-like and ST30-like parental backgrounds are known to have existed as methicillin-susceptible isolates during the 1950s [71], but we have been unable to locate thus far any bona fide methicillin-susceptible ST239 isolates from any time period. If such isolates could be located, it would be important to determine whether they represented losses of SCC*mec* type III or susceptible ancestors.

We discovered that the *ccrB* and *dru* loci, not accessory components, constituted phylogenetically informative markers for SCC*mec* type III. If the purpose of SCC*mec* typing is to identify related variants, then our results call for a re-evaluation of the popular approach of targeting accessory components in subtyping assays [45], [72], [73]. The phylogenetic utility of these markers in other SCC*mec* types should be investigated. Since the accessory components of SCC*mec* type III encode resistances to a broad array of antimicrobials, these markers may still be of epidemiological relevance, but their use for establishing relatedness of ST239 isolates outside of local outbreak situations is not warranted.

## Supporting Information

Text S1 Supplemental methods.
(0.04 MB DOC)

Table S1 Characteristics of study isolates.
(0.08 MB XLS)

Table S2 Additional primer sets.
(0.04 MB XLS)

Figure S1 Characteristics of the discovery panel of isolates. PFGE was done with standard methods. Table lists strain name, geographic source and date of isolation, haplotype defined from 32 loci, SCCmec multiplex PCR type and ccrB:dru sequence type.
(0.43 MB TIF)

Figure S2 SCCmec type III variants as detected by the multiplex PCR assay. (A) Map shows relative locations of select loci, not to scale. (B) Table lists presence or absence of multiplex PCR products using primers from reference 45. ccrC was also amplified, independently, using primers from reference 49. Patterns for types III–IIIB are described in reference 45. Types IIIC–IIIG are named solely for communication purposes here. Type IIIG corresponds to the unnamed type described by reference 50. (C) Example of multiplex PCR types III–IIIG from the discovery panel of isolates. Lanes 1 and 12, 100 bp ladder (Promega); lane 2, ANS46 (III); lane 3, HUSA304 (III); lane 4, 2HK (IIIH); lane 5, HU25 (IIIA); lane 6, HDG2 (IIIB); lane 7, PolJ405 (IIIC); lane 8, Fin98541 (IIID); lane 9, DAR20 (IIIG); lane 10, DEN907 (IIIF); lane 11, K03–030 (IIIE).
(0.43 MB TIF)
