# Supplementary material for: Population Structure of a Hybrid Clonal Group of Methicillin-Resistant Staphylococcus aureus, ST239-MRSA-III
Source: PLoS One. 2010 Jan 5;5(1):e8582. doi: 10.1371/journal.pone.0008582 (PMC2797301; doi:10.1371/journal.pone.0008582)
Supplement: Text S1 — Supplemental methods. (0.04 MB DOC) [file pone.0008582.s001.doc]

**Reduced representation shotgun sequencing (RRS)**

Genomic DNA was isolated from the discovery panel of isolates with the Easy-DNA kit (Invitrogen). An equimolar mixture of these DNA preparations was digested to completion with *Rsa*I (New England Biolabs), and 100 µg of the digested mixture was separated by electrophoresis in a 1.5% agarose gel. A narrow, two mm band adjacent to the 500-600 bp bands of a DNA size ladder was excised from the gel and purified using the Wizard SV Gel and PCR Clean-up System (Promega). The fragments were dA-tailed using the Single dA Tailing Kit (Novagen), ligated to pCRII (Invitrogen), and transformed into chemically-competent Top10 *E. coli* (Invitrogen), as per the manufacturers' instructions. Clones containing plasmids with inserts were identified with blue-white screening. Based on the genome-sequenced *S. aureus* strain MRSA252 [1], the *S. aureus* chromosome is expected to have 438 unique *Rsa*I fragments in the 500-600 bp range. Here, sequences from 457 clones were obtained after PCR amplification of their inserts. A much larger library would have been needed to guarantee that all targeted *Rsa*I fragments from all 10 diverse isolates of the discovery panel were included (i.e. full depth). Our reduced representation shotgun library produced 153 unique non-overlapping contigs (i.e. loci), including 77 multi-sequence contigs (i.e. counted as screened loci) and 76 single-sequence contigs. Of the 77 multi-sequence contigs, 12 contained variation and, after excluding paralogous loci such as rRNA clusters, four loci were selected. Of the 76 single-sequence contigs, one locus was selected based on polymorphisms that were discovered through comparisons with unfinished ST239 genome-sequencing projects. Finally, sequences from these five loci were obtained for all 111 isolates.

Ascertainment methods such as RRS can provide an efficient approach to the discovery of genetic variation among closely related isolates; however, they can also introduce bias into population genetic and phylogenetic analyses. Briefly, ascertainment can cause an excess of higher frequency alleles and a scarcity of lower frequency alleles [2], and it can cause branch collapse along the tree pathways that connect the discovery panel of isolates [3]. In geographically structured populations, ascertainment bias is largest when the discovery panel is geographically limited [4]. The 10 diverse isolates of the discovery panel used here were selected to maximize diversity based on PFGE and SCC*mec* data, geography, and date of isolation. This panel included multiple prototype ST239 variants. Characteristics of the discovery panel are listed in Figure S1 adjacent to their PFGE patterns.

**Details of data analyses**

The Slatkin-Maddison (SM) test of population structure involved mapping a character of interest (e.g. geographic source of isolation) onto the ST239 phylogeny in a manner that minimized the number of changes of its character states (e.g. different continents for a geographic character). The biological interpretation of these changes depends on the character (e.g. changes represent migration events for a geographic character). Tree length was the objective measure used to choose between the many different possible reconstructions of the character on the tree, and it equals the sum of the cost of all changes. Unordered characters had a cost of one for all changes and were reversible (e.g. Europe-to-Asia changes cost the same as Asia-to-Europe changes). Date of isolation was treated as an ordered character, and a step matrix was used to reflect the cost of change between character states; the cost was the number of years between dates and was irreversible. Since the ST239 phylogeny contained unresolved nodes, an average tree length for each character was calculated over 1000 random resolutions of the tree; this statistic was called *S_RR_*. The statistical significance of *S_RR_* values were tested against null distributions of tree lengths constructed, under the null hypothesis of no association between the ST239 phylogeny and the character of interest, by 1000 random splitting-joining replicates.

The subpopulation differentiation (SD) test of population structure used the *K_ST_* statistic. *K_ST_* measures between-subpopulation haplotype diversity where, by analogy to the parsimony approach above, a character of interest represents a population and its character states represent subpopulations. *K_ST_* ranges from 0 to 1, and increases as subpopulations acquire distinction in haplotypes. *K_ST_* makes use of both the frequency with which haplotypes occur and the number of nucleotide differences between their sequences. The statistical significance of *K_ST_* values were tested against null distributions of *K_ST_* values constructed, under the null hypothesis of no subpopulation differentiation, by 1000 random shufflings of haplotypes between subpopulations. We also used the average number of pairwise nucleotide differences between countries, *K_xy_*, with the neighbor-joining algorithm to visualize geographic differentiation; for this analysis we included all isolates from the 22 countries with >1 sampled isolate.

Regression analyses between tree root-to-haplotype distances and haplotype dates of isolation was used to further study temporal population structure. For these analyses, the tree was constructed with the neighbor-joining algorithm from a matrix of *p*-distances, and was rooted at the node most compatible with a molecular clock. Estimates of the rate of evolution and date of the most recent common ancestor (MRCA) were made from the slope and x-intercept, respectively, of the regression line [5]. Bias in these crude estimates due to well-sampled haplotypes with many different dates of isolation was investigated using a jackknife procedure that sequentially dropped each haplotype; the delete-*mj* jackknife [6] was performed to account for unequal numbers of dates of isolation among haplotypes. Jackknifing provided haplotype bias-corrected estimators, *θ_mj_*, of the rate of evolution and date of the MRCA and their variances, Var(*θ_mj_*), following the formulae of Busing et al. [6]. These data were used to construct 95% confidence intervals with the formulae: *θ_mj_* +/- 2 √Var(*θ_mj_*).

Over-sampled haplotypes due to epidemiological clustering introduce artifacts into analyses of bacterial population structure [7]. Thus, for each different haplotype that occurred with a different character state or subpopulation, only a single isolate was used in the SM, SD, and regression analyses outlined above. Without this "clone-correction", nearly every analysis falsely achieved high levels of statistical significance. Two additional issues were addressed in an attempt to more accurately estimate the rate of evolution and date of the MRCA. First, the ascertainment methods involved a selection process to identify variable loci among ST239 isolates, so it was expected that these loci would result in more nucleotide changes per site than occur in the typing loci which were not selected based on their variation among ST239 isolates. Indeed, analyses of contingency tables showed that the ascertained loci discovered significantly (Fisher's exact test, *P*=0.03) more changes per site than the typing loci. Thus, homogeneous "dummy" DNA was added to the ascertained loci to make them comparable (Fisher's exact test, *P*=1.0) to the typing loci in terms of changes per site, thereby crudely adjusting for the inflationary effects of ascertainment. Secondly, ST239 is a hybrid, so different portions of its chromosome have different ancestors. Concordance among estimates made from separate parental portions of the chromosome can provide evidence that the isolates shared a single common ancestor [8]. Thus, dummy DNA was added separately to the ST8- and ST30-like loci, and separate estimates of the rate of evolution and date of the MRCA were made.

**References for supplemental methods**

1. Holden MT, Feil EJ, Lindsay JA, Peacock SJ, Day NP, et al. (2004) Complete genomes of two clinical *Staphylococcus aureus* strains: evidence for the rapid evolution of virulence and drug resistance. Proc Natl Acad Sci USA 101: 9786-9791.
2. Nielsen R, Hubisz MJ, Clark AG (2004) Reconstituting the frequency spectrum of ascertained single-nucleotide polymorphism data. Genetics 168:2373-2382.
3. Pearson T, Busch JD, Ravel J, Read TD, Rhoton SD, et al. (2004) Phylogenetic discovery bias in *Bacillus anthracis* using single-nucleotide polymorphisms from whole-genome sequencing. Proc Natl Acad Sci USA 101: 13536-13541.
4. Rosenblum EB, Novembre J (2007) Ascertainment bias in spatially structured populations: a case study in the eastern fence lizard. J Hered 98: 331-336.
5. Drummond A, Pybus OG, Rambaut A (2003) Inference of viral evolutionary rates from molecular sequences. Adv Parasitol 54: 331-358.
6. Busing FM, Meijer E, Van Der Leeden R (1999) Delete-m jackknife for unequal m. Statistics and Computing 9: 3-8.
7. Maynard Smith J, Smith NH, O'Rourke M, Spratt BG (1993) How clonal are bacteria? Proc Natl Acad Sci USA 90: 4384-4388.
8. Tee KK, Pybus OG, Parker J, Ng KP, Kamarulzaman A, et al. (2009) Estimating the date of origin of an HIV-1 circulating recombinant form. Virol 387: 229-234.
