# Supplementary figures and images for: Population Structure of a Hybrid Clonal Group of Methicillin-Resistant Staphylococcus aureus, ST239-MRSA-III
Source: PLoS One. 2010 Jan 5;5(1):e8582. doi: 10.1371/journal.pone.0008582 (PMC2797301; doi:10.1371/journal.pone.0008582)

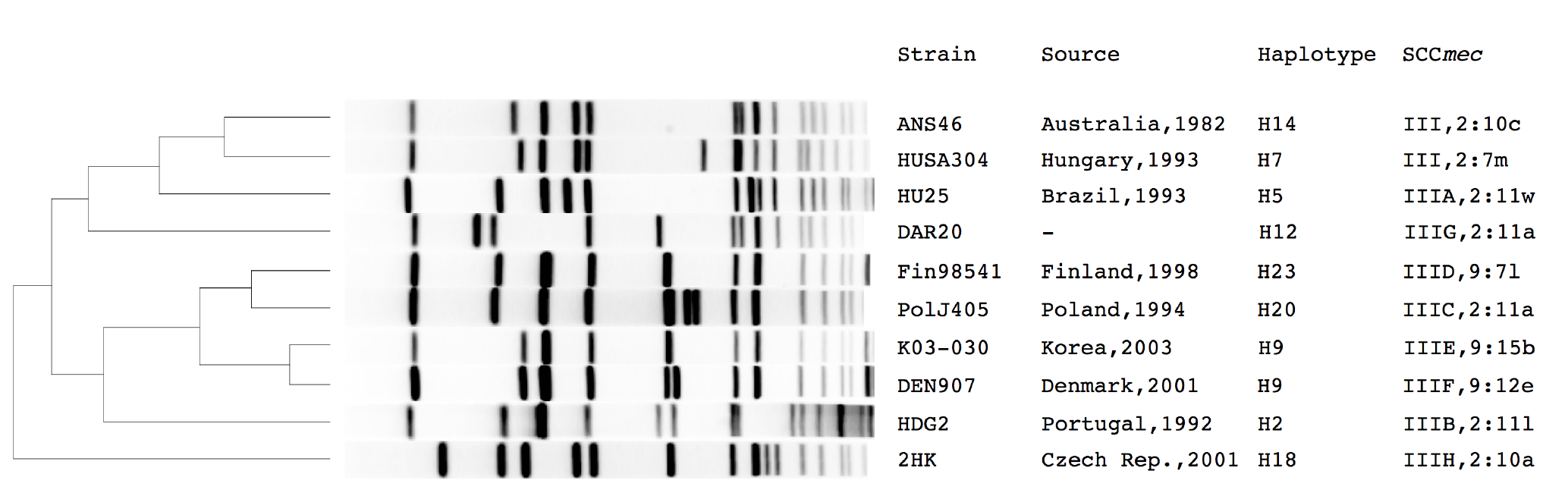

Supplement: Figure S1 — Characteristics of the discovery panel of isolates. PFGE was done with standard methods. Table lists strain name, geographic source and date of isolation, haplotype defined from 32 loci, SCCmec multiplex PCR type and ccrB:dru sequence type. (0.43 MB TIF) [file pone.0008582.s004.tif]

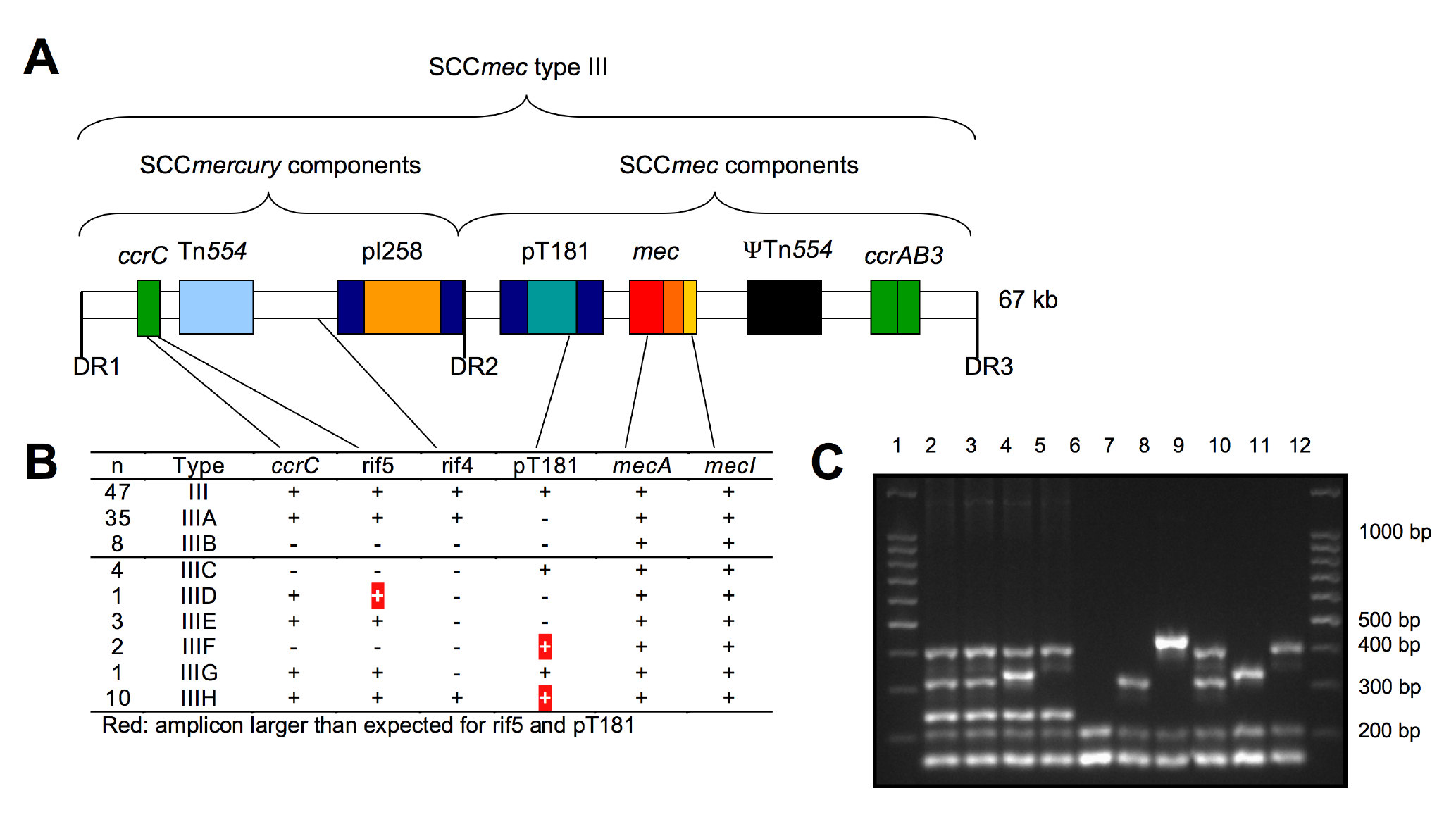

Supplement: Figure S2 — SCCmec type III variants as detected by the multiplex PCR assay. (A) Map shows relative locations of select loci, not to scale. (B) Table lists presence or absence of multiplex PCR products using primers from reference 45. ccrC was also amplified, independently, using primers from reference 49. Patterns for types III–IIIB are described in reference 45. Types IIIC–IIIG are named solely for communication purposes here. Type IIIG corresponds to the unnamed type described by reference 50. (C) Example of multiplex PCR types III–IIIG from the discovery panel of isolates. Lanes 1 and 12, 100 bp ladder (Promega); lane 2, ANS46 (III); lane 3, HUSA304 (III); lane 4, 2HK (IIIH); lane 5, HU25 (IIIA); lane 6, HDG2 (IIIB); lane 7, PolJ405 (IIIC); lane 8, Fin98541 (IIID); lane 9, DAR20 (IIIG); lane 10, DEN907 (IIIF); lane 11, K03–030 (IIIE). (0.43 MB TIF) [file pone.0008582.s005.tif]
